# Evaluation of an Electro-Pneumatic Device for Artificial Capillary Pulse Generation used in a Prospective Study in Animals for Surgical Neck Wound Healing

**DOI:** 10.1038/s41598-019-46397-0

**Published:** 2019-07-08

**Authors:** J. Foltyn, A. Proto, D. Oczka, R. Halfar, T. Klinkovsky, L. Skoloudik, M. Cerny, V. Chrobok, A. Ryska, V. Radochova, M. Litschmannova, M. Penhaker, J. Mejzlik

**Affiliations:** 1 Special Medical Technology Co., Ltd., Prague, Czech Republic; 2 Department of Cybernetics and Biomedical Engineering, VSB - Technical University of Ostrava, Ostrava, Czech Republic; 3 Department of Otorhinolaryngology and Head and Neck Surgery, University Hospital Hradec Kralove, Charles University, Faculty of Medicine in Hradec Kralove, Hradec Kralove, Czech Republic; 4 Fingerland’s institute of Pathology, University Hospital Hradec Kralove, Charles University, Faculty of Medicine in Hradec Kralove, Hradec Kralove, Czech Republic; 5 Vivarium, Faculty of Military Health Sciences, University of Defence, Brno, Czech Republic; 6 Department of Applied Mathematics, VSB - Technical University of Ostrava, Ostrava, Czech Republic

**Keywords:** Carotid artery disease, Outcomes research, Biomedical engineering

## Abstract

The paper examines the development and testing of an electro-pneumatic device for wound healing therapy after surgery in the neck area. The device generates air pressure values in a miniaturized cuff using electronic circuitry to drive an electro-valve and air compressor. The device works in two distinct modes: continuous pressure mode and pulsating pressure mode. The pressure value setting can vary from 3 to 11 mmHg, and the pulsating pressure mode’s operating frequency range is approximately 0.1 to 0.3 Hz. Laboratory measurements were conducted to evaluate the device’s correct functioning in both continuous and pulsating pressure modes. A four-day prospective study with animals (*n* = 10) was also conducted to evaluate neck wound healing therapy using the electro-pneumatic device. Out of the twelve histological parameters analysed to reveal the differences between the experimental and control wounds, only one demonstrated a significant difference. Out of the ten animals treated with the device, three showed a significant difference in terms of benefit after therapy. We can therefore conclude that the device potentially improves the wound healing process in the neck area if the pre-set air pressure value does not exceed 8 mmHg.

## Introduction

Wound healing is the biological process for successful regeneration of skin tissue. It occurs through four established biological processes: haemostasis, inflammation, proliferation and remodelling. Correct wound healing is achieved by these phases overlapping, which occurs in a precise and continuous time-sequence^1^.

Wounds are categorized into two groups: *acute* and *chronic*. The causes of acute wounds can be surgical incisions, trauma, burns and abrasions, whereas those for chronic wounds may be slow onset, disease and recurrent injuries. Acute wounds usually heal within three weeks. However, chronic wounds may occur if the wound healing process fails^2,3^. Prolonged healing time leads to disadvantages in the health care system, particularly in terms of costs for wound care products and related medical treatments^4,5^. The most important goal in the wound healing process is therefore prompt closure of the wound and avoiding an acute wound becoming a chronic wound.

The *Wound Healing Society* (WHS) lists eleven categories of physical and chemical barriers that can alter the wound healing process^6^. These categories are further sub-divided into two groups: (1) *local* (wound perfusion, tissue viability, haematoma and/or seroma, infection and mechanical factors) and (2) *systemic* (immunology, oncology, miscellaneous systemic conditions, thermal injuries, external agents and excessive scarring).

The first of the eleven categories, *wound perfusion*, necessitates adequate blood perfusion as a physiological condition for a successful wound healing process. In fact, the perfusion phenomenon permits the interaction between blood and tissue, including the supply of nutrients and oxygen and the removal of harmful elements, such as toxins and CO_2_. This interaction occurs in the microvascular network at the level of capillaries^7^.

The occurrence of a wound inevitably changes the rate and the extent of perfusion in the capillary bed, altering the proper exchange of biological and chemical substances between blood and tissue. Indeed, poor local perfusion increases the risk of wound infection and may generate tissue ischemia and necrosis due to the lack of nutrients and oxygen. Conversely, excess perfusion may form severe oedema in tissues and cause bleeding^8^. Therefore, adequate capillary perfusion is a key factor in obtaining a quick and satisfactory wound healing process without complications.

Dressings using sterile compression bandages protect wounds from infection and improve capillary perfusion. However, manual placement of these bandages can be harmful to patients if a too restrictive or insufficient covering is applied, which can lead to tissue damage^9^.

Again, it is important to monitor vital tissue parameters such as oxygen, temperature, pH, enzymes and bacteria^10–14^. Many new systems have been developed for this purpose, including flexible sensors for wound management, multifunctional skin-like electronics for clinical monitoring and smart bandage technologies for wound diagnostics^15–19^. They are non-invasive and can be applied directly to the skin or inside a bandage to monitor the physiological characteristics of the wound. Although these systems are good detectors of the parameters indicating the proper healing process, they are not able to regulate the pressure values related to blood perfusion in the capillary bed.

Figure S1 (see Supplementary Material) shows the attenuation of the amplitude of blood pressure oscillation as blood passes from the arteries to the capillaries. Each tissue has its own microvascular system, which varies between the different body organs. It is important therefore to know the physiological characteristics of the affected organ in order to achieve proper care during the healing process.

Our research focused on the healing of surgical wounds in the neck area. In this part of the body, the correct postoperative pressure on tissue should be no more than approximately 10 mmHg. This limit represents the value of the postoperative pressure for detecting wound re-bleeding^20,21^ and in recognizing neck compartment syndrome^22^. Similarly, the value of capillary pulse pressure amplitude ranges from approximately 0.5 to 12 mmHg^23,24^. In this work, we present the development of an electro-pneumatic device able to provide soft mechanical pressure on surgical wounds in the neck area in order to assist wound perfusion by generating artificial capillary pulses in damaged tissue. This electro-pneumatic device generates a pulse pressure amplitude range of 3 to 11 mmHg and can operate in both continuous and pulsating pressure modes.

## Methods

### Electro-pneumatic device

The device has three main parts: (1) a disposable pressure cuff, (2) electronics comprising analogue and digital circuits to control and acquire air pressure signals in the cuff and (3) a battery module to power the device.

**Figure 1:**
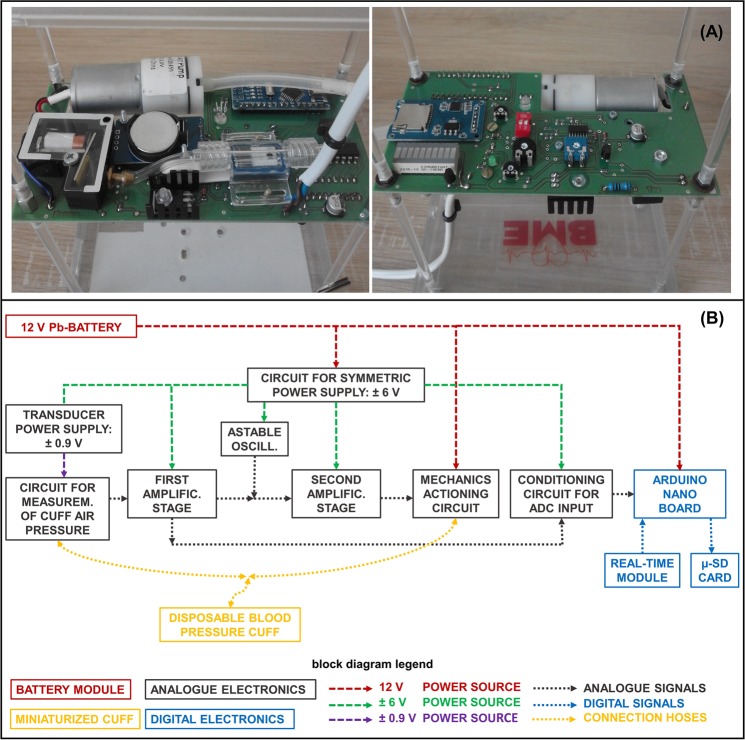
Electronic circuit (**A**) and block diagram of the complete system (**B**).

Figure 1A illustrates the electronic circuitry and Fig. 1B shows a block diagram of the complete system.

The cuff is a disposable blood pressure NEONATE 5, which has sizes of 8 to 15 cm. The small size guarantees correct placement in the neck area. The pressure hose connections and connection point in the electronic unit have an internal-external diameter of 3 to 6 mm. The electronic circuit regulates, senses and records the cuff’s air pressure values.

The first block of electronics is a symmetric circuit for the voltage supply. The design of a virtual ground converts the 12 V battery output into a dual voltage supply (i.e. ±6 V). A green 1.8 V LED indicates when the system is switched on. It also serves as a reference for stable voltage (i.e. ±0.9 V), in supplying energy to the Hospira Transpac IV transducer, which administers pressure values in the cuff.

The transducer is configured in a Wheatstone bridge circuit, whose output leads towards a double amplification stage. The output of the first amplification stage is a voltage signal proportional to the value of the measured air pressure and displaced by an offset settled by the value of a trimmer. This trimmer sets the minimum pressure value in the cuff. The second amplification stage controls actuation of both the electro-valve and air compressor according to the pre-set value, which ranges from 3 to 11 mmHg. Therefore, if the value of the measured pressure after the first amplification stage is less than the pre-set value, the second amplification stage provides the gain to properly drive the mechanical part of the circuit.

A power transistor switches on the air compressor, while an amplifier transistor controls functioning of the electro-valve. The electro-valve is also equipped with a capacitor and a Schottky diode. These components make sure the valve is properly switched on even after the air compressor has been switched off.

To generate pulsating pressure, an astable oscillator circuit generates a rectangular waveform. The operation frequency can be regulated from 0.1 to 0.3 Hz over a time-period of approximately 3.3 to 9 s.

An Arduino Nano board with a microSD card slot and real-time clock module records and displays the acquired pressure data. The output signal first passes through a differential amplifier in the signal conditioning stage. A 10-bit A/D converter then samples the pressure data at a sampling rate of 4 Hz, with a 5 V reference voltage. Finally, the data is stored on a microSD card as a.csv file. The file’s name takes the form “YYMMDD_HHMMSS”, indicating exactly when measurement began because of the use of the real-time clock module. A CR2032 battery powers the real-time module separately; replacing the battery of the whole system does not therefore affect the device’s clock.

Continuous operation of the active mechanical components (electro-valve and air compressor) means the power consumption of the system is high overall (see Table S1). A 12 V Pb-battery was therefore selected to power the whole system. This battery is a valve regulated lead-acid (VRLA) battery, maintenance-free and equipped with a pressure relief valve to remove excess gas. The main difference between this maintenance-free battery and a conventional Pb-battery is in its technology: the electrolyte is not in a liquid state but bonded in a glass fibre mat (AGM). The battery can thus supply a lower current over a longer period of functioning.

To check the battery’s condition during measurements, digital electronics monitor its voltage levels and show the status on an RGB LED. When the LED is red, the battery requires charging. Figure S2 (see Supplementary Material) shows the battery-discharge curve with corresponding voltage values when the LED light changes colour.

### Calibration procedure

Six electro-pneumatic devices we developed. The performance of each device was influenced by the electronic and mechanical components that distinguished the entire system and by the parameters of the A/D converter on the Arduino Nano board. It is important to mention that the circuit driving the electro-valve and air compressor is analogue, while the measured air pressure values are recorded through a signal passing through an analogue to digital converter.

In the analogue circuit driving the mechanical components, the system’s precision is limited by the inertia of the response to the continuous pressure increase (i.e. the compressor) and the discontinuous pressure drop (i.e. opening of the electro-valve). A non-linear regulating loop also crosses a section of the nozzle (i.e. the valve outlet port). In the data acquisition circuit, the Hospira Transpac IV transducer measures the air pressure in the cuff. The measured value is compared to a voltage value that is either continuous (i.e. “DC” value), or pulsating (i.e. “DC + AC” value). The signal then passes through the conditioning circuit before reaching the input of analogue to digital converter.

The response of the calibration curve in each device is shown in Fig. S3 (see Supplementary Material). To achieve these curves, a pressure meter (DPM2Plus pressure meter) was added to the measurement chain. Therefore, by applying known pressure values (*ΔP*_*in*_ = 1 mmHg) from 1 mmHg to 12 mmHg in the system and digitally measuring the voltage output through the Arduino Nano board, several discrete values were collected. The conversion coefficient was then derived through interpolation and approximation of the previously acquired discrete values. The curves show a linear tendency, with offset values that can be ignored. The values of sensitivity and resolution parameters were 0.22 V/mmHg and 0.11 mmHg, respectively (see Supplementary Appendix A).

### Surgical procedure

The “Scientific Committee on Experimental Animal Welfare” at the Faculty of Military Health Sciences, University of Defence, Brno, approved all of the steps of the prospective study with animals in protocol ID: ET HIC Number 50-1/2016-684800. The surgical procedures were conducted in accordance with the relevant guidelines and regulations^25^.

Ten pigs were used as test animals for the *in-vivo* experiments. Two identical surgical wounds were made on the upper chest of each pig, one being the experimental wound, the other the control wound. The surgical wounds were acute wounds. A surgeon made an incision in the skin and subcutaneous tissue up to the muscle layer. A muscle injury was also created by excision of the muscle corresponding to the base of the wound. The size of the final wound was approximately 14 cm^2^, in the shape of a leaf. The wounds were then closed using the mattress suture technique. The surgeon applied six sutures spaced approximately 1 cm from each other.

An adhesive patch approximately 20 cm × 10 cm in size was then applied to cover each wound. For the experimental wound, a cuff was inserted into the bandage, with its hoses kept outside the dressing. Another bandage was again applied to both the experimental and control wounds. Finally, a waterproof pouch was attached to the chests of all the pigs to maintain the entire system in a stable position.

The electro-pneumatic device was only applied to the experimental wound. The experiment was conducted over four days following surgery, and on the fifth day, the pigs were euthanized in order to proceed with a histological analysis.

### Parameters for the histological analysis

Two equal specimens representing the experimental and control wounds were excised from each pig. These specimens enclosed the cut skin area with its subcutaneous tissue and the surrounding area of approximately 2 cm. In total, 20 specimens were collected, 10 of the experimental wounds and 10 corresponding to the control wounds. A random four-digit number was assigned to each specimen and all specimens were sent to a pathologist^25^. The specimens’ examinations were conducted blindly to reduce bias. The physician was, in fact, unaware of what the experimental or control specimens were.

The pathologist studied the specimens, analysing the following parameters:

- abscesses (*abs*.), which indicate the tissue’s reaction to prevent the spread of infection,
- phlegmon (*phl*.), which is an inflammatory process in the formation of purulent exudate, related to bacterial infection,
- bacteria (*bact*.) colonizing the wound on the skin’s surface, in the sinus tract and deep in the abscess cavity,
- sinus tract granulation (*s*. *t*. *gr*.) along the vertical course of the wound and the relative granulation depth (*gr*. *depth*),
- re-epithelization of the tissue (*reepith*.), which indicates restoration of the epithelium,
- the cavity’s diameter (*cav*. *diam*.), which indicates the size of the residual cavity inside the wound,
- septal cell proliferation (*s*. *c*. *p*.) in the proximity of the cavity and radially into the surrounding fat, (i.e. septae of fat lobules),
- sequestration (*seq*.), which indicates the presence of necrotic tissue and the maximum size of the sequester diameter (*seq*. *diam*.),
- the wound closure tract (*w*. *c*. *t*.) and basophilic muscle decomposition (*b*. *m*. *d*.), which provide evidence about damage to the muscle fibre.

To conduct the histological analysis, the pathologist assigned quantitative discrete values to each parameter, except for *cav*. *diam*. and *seq*. *diam*. parameters, which were assigned continuous values (see Supplementary Table S2). The discrete values assigned to the histological parameters were 0 to 3, where “0” indicated the absence of the checked parameter, “1” indicated a minor occurrence, “2” a moderate occurrence and “3” a major occurrence of the parameter. In describing the parameters’ features, it is important to note that a discrete value of “0” indicated the best wound healing process in the *abs*., *phl*., *bact*., *s*. *c*. *p*. and *seq*. parameters, and conversely, a discrete value of “3” indicated the best wound healing process for the *s*. *t*. *gr*., *gr*. *depth*, *reepith*., *w*. *c*. *t*. and *b*. *m*. *d*. parameters. The assigned values for the *cav*. *diam*. and *seq*. *diam*. parameters were given in millimetres.

A Wilcoxon signed-rank test (α = 0.05) was performed to reveal the differences between the experimental and control specimens in terms of the analysed histological parameters. A significant difference was considered if the *p*-value was less than 0.05. A descriptive statistic was also performed because of the narrow range of discrete values assigned to the specimens (i.e. 0 to 3) and the low sample number considered in the Wilcoxon signed rank-test (*n* = 10). The descriptive statistic was performed on the discrete values using contingency tables (see Supplementary Tables S3–S12), and for the analysis of continuous values, the values assigned to the experimental and control specimens were subtracted. The median and interquartile range were then calculated as measure of variability (see Supplementary Tables S13, S14). The pathologist stated that a visible improvement could be seen if the calculated difference was at least 5 mm in these continuous values.

To conclude the histological analysis, another Wilcoxon signed-rank test (α = 0.05) was performed to reveal the statistical results for all the animals. Each animal corresponded to a single test. A significant difference was considered if the *p*-value was less than 0.05. Table S15 (see Supplementary Material) shows the discrete values assigned to the histological parameters, where “0” indicated the occurrence of the best wound healing process while “3” represented the worst.

## Results

### Performance of the electro-pneumatic device

The electro-pneumatic device can work in two distinct modes: continuous pressure mode and pulsating pressure mode. The user selects the device’s operating modes using three controllers. The first is a switch to select the device’s continuous or pulsating operating mode. The user then sets the pressure value in the range 3 to 11 mmHg using a potentiometer. When the device is used in pulsating mode, another potentiometer allows the value of the operating frequency to be set in the range 0.1 to 0.3 Hz. When this mode is used, the level of pressure in the cuff should ideally decrease to 70% of the pre-set value.

To verify the performance of the electro-pneumatic device, the continuous and pulsating operation modes were tested in the laboratory. Five pre-set pressure values of approximately 3.6, 5.2, 6.9, 8.5 and 10.5 mmHg were used to evaluate continuous mode. Operation frequencies of approximately 0.11, 0.13, 0.16, 0.20 and 0.28 Hz were used to assess pulsating mode. Figure S4 (see Supplementary Material) shows the relationship between the slider positions of the potentiometers and the pre-set values for air pressure and operating frequency.

Table 1 summarizes the results of the laboratory tests. All the acquired data were analysed with *Matlab software by MathWorks*, *Inc*., using the *MATLAB boxplot(X)* function to determine the minimum and maximum values, the upper and lower whiskers, the median and the 25^th^ and 75^th^ percentile values. The 25^th^ percentile is the middle value between the lowest value and the median value, while the 75^th^ percentile is the middle value between the median and the highest value. The distance between the 75^th^ and 25^th^ percentiles is called the interquartile range. Whiskers were drawn from the ends of this interquartile range to the furthest observations in the whisker length. These were the limits beyond which the pressure values were considered outliers. Outliers were values more than 1.5 times the value of the interquartile range.

**Table 1:** Results of the electro-pneumatic device’s performance in the laboratory tests.

	Total time (s)	Operation frequency (Hz)	Min. value (mmHg)	Max. value (mmHg)	Lower whisker (mmHg)	Upper whisker (mmHg)	Median value (mmHg)	25^th^ percentile value (mmHg)	75^th^ percentile value (mmHg)	Pressure decrease (%)	Was the test successful?
*pres*. *value n*° *1*	615	—	3.38	4.94	3.56	3.69	3.62	3.61	3.64	99	YES
*pres*. *value n*° 2	611	—	3.62	10.35	4.58	5.65	5.11	4.97	5.23	95	YES
*pres*. *value n*° 3	641	—	4.56	10.02	6.14	7.38	6.75	6.61	6.91	96	YES
*pres*. *value n*° 4	610	—	4.98	11.83	7.38	8.47	7.91	7.77	8.04	97	YES
*pres*. *value n*° 5	619	—	6.84	19.68	9.58	11.02	10.28	10.11	10.47	97	YES
***Press***. ***value**** n*° ***1***											
*freq*. *of puls*. *n*° *1*	607	0.11	3.33	5.78	3.33	3.80	3.57	3.48	3.61	96	NO
*freq*. *of puls*. *n*° 2	609	0.13	3.34	5.65	3.34	3.84	3.59	3.48	3.62	96	NO
*freq*. *of puls*. *n*° 3	625	0.16	3.33	5.87	3.33	3.90	3.58	3.47	3.63	96	NO
*freq*. *of puls*. *n*° 4	611	0.20	3.35	5.91	3.35	3.85	3.59	3.50	3.64	96	NO
*freq*. *of puls*. *n*° *5*	619	0.28	3.33	5.74	3.36	3.85	3.61	3.52	3.64	97	NO
*Mean values*	—	—	3.34	5.79	3.34	3.85	3.59	3.49	3.63	96	—
***Press***. ***value**** n*° ***2***											
*freq*. *of puls*. *n*° *1*	604	0.11	3.38	7.84	3.38	6.78	4.65	3.84	5.02	76	YES
*freq*. *of puls*. *n*° 2	604	0.13	3.40	7.92	3.40	6.77	4.75	3.87	5.10	76	YES
*freq*. *of puls*. *n*° 3	605	0.16	3.39	7.98	3.39	7.02	4.79	3.81	5.10	75	YES
*freq*. *of puls*. *n*° 4	662	0.20	3.36	7.89	3.36	7.05	4.55	3.75	5.07	74	YES
*freq*. *of puls*. *n*° *5*	605	0.28	3.36	7.79	3.36	7.42	4.89	3.87	5.35	72	YES
*Mean values*	—	—	*3*.*38*	*7*.*88*	*3*.*38*	*7*.*01*	*4*.*73*	*3*.*83*	*5*.*13*	75	—
***Press***. ***value**** n*° ***3***											
*freq*. *of puls*. *n*° *1*	605	0.11	3.69	9.57	3.69	9.42	6.42	5.07	6.82	74	YES
*freq*. *of puls*. *n*° 2	609	0.13	3.48	9.61	3.48	9.36	6.39	5.10	6.81	75	YES
*freq*. *of puls*. *n*° 3	605	0.16	3.49	9.56	3.49	9.56	6.58	5.12	6.93	74	YES
*freq*. *of puls*. *n*° 4	608	0.20	3.50	9.59	3.50	9.59	6.43	5.07	6.96	73	YES
*freq*. *of puls*. *n*° *5*	606	0.28	3.56	9.74	3.56	9.55	6.68	5.34	7.03	76	YES
*Mean values*	—	—	*3*.*54*	*9*.*61*	*3*.*54*	*9*.*50*	*6*.*50*	*5*.*14*	*6*.*91*	74	—
***Press***. ***value**** n*° ***4***											
*freq*. *of puls*. *n*° *1*	610	0.11	3.94	11.94	3.94	11.31	8.13	6.47	8.49	76	YES
*freq*. *of puls*. *n*° 2	603	0.13	4.08	11.89	4.08	11.60	7.96	6.40	8.50	75	YES
*freq*. *of puls*. *n*° 3	602	0.16	4.12	12.09	4.12	11.65	8.13	6.38	8.54	75	YES
*freq*. *of puls*. *n*° 4	603	0.20	4.27	13.80	4.27	11.73	8.20	6.43	8.70	74	YES
*freq*. *of puls*. *n*° *5*	604	0.28	4.14	11.99	4.14	11.99	8.40	6.73	8.85	76	YES
*Mean values*	—	—	*4*.*11*	*12*.*34*	*4*.*11*	*11*.*66*	*8*.*16*	*6*.*48*	*8*.*62*	75	—
***Press***. ***value**** n*° ***5***											
*freq*. *of puls*. *n*° *1*	604	0.11	3.61	14.42	5.46	13.65	9.79	8.28	10.45	79	YES
*freq*. *of puls*. *n*° 2	606	0.13	5.91	14.68	5.91	13.87	10.02	8.27	10.50	79	YES
*freq*. *of puls*. *n*° 3	601	0.16	5.69	14.05	5.69	14.05	9.98	8.13	10.56	77	YES
*freq*. *of puls*. *n*° 4	604	0.20	5.68	13.99	5.68	13.99	10.15	8.04	10.77	75	YES
*freq*. *of puls*. *n*° *5*	606	0.28	5.87	16.53	5.87	15.13	10.23	8.17	10.96	75	YES
*Mean values*	—	—	*5*.*35*	*14*.*73*	*5*.*72*	*14*.*14*	*10*.*03*	*8*.*18*	*10*.*65*	77	—

Table 1 also shows the percentage values at the decreased pressure level when the device was set in pulsating operation mode. This percentage value was calculated between the 75^th^ and 25^th^ percentile values. In the final column, “YES” indicated successful performance of the device, while “NO” indicated an unsuccessful test.

All the tests performed indicated by the results in Table 1 were conducted for at least 10 minutes. In pulsating operation mode, the decrease of the air pressure signal to 70% of the initially pre-set value did not occur in the tests indicated under “*Press*. *value no*. *1*”, which was a result of lower saturation limits, which are determined by the electronic circuitry.

All the other performed tests confirmed that the device functioned successfully. Even when the air pressure did not decrease to 70% of the pre-set value, we calculated a mean value of approximately 75%.

**Figure 2:**
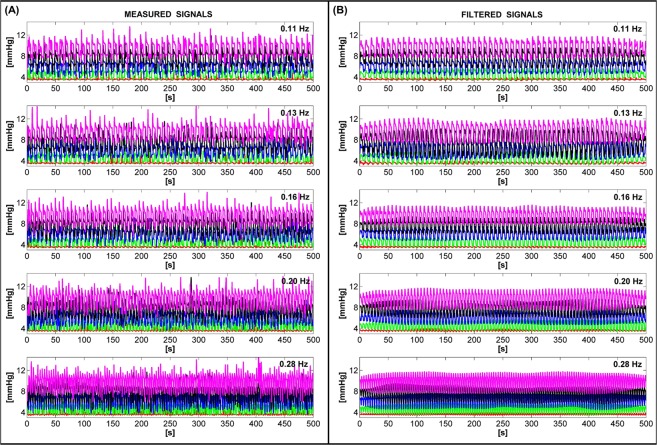
The measured (**A**) and filtered (**B**) air pressure signals for tests performed in pulsating operation mode.

Figure 2 shows the measured (Fig. 2A) and filtered (Fig. 2B) signals for tests performed in pulsating operation mode. Figure 2A clearly shows numerous peaks exceeding the pre-set air pressure values in the signal wave-fronts. This drawback is because of the electro-valve’s switching circuit behaviour. The circuit does not react instantly, the minimum time to switch on or switch off being a second. The measured air pressure values in the cuff show that at each second the switching circuit was either recharged for the next second or not recharged. When switching occurred, the measured air pressure signal showed unwanted peaks (i.e. a measurement uncertainty of approximately ±1 mmHg). Conversely, Fig. 2B shows the filtered signals as ideal signal wave-fronts. Signal filtering was performed digitally using *Matlab software by MathWorks*, *Inc*. (see Supplementary Appendix B).

### Acquisition of the air pressure signals during the prospective study with animals

Ten pigs were subjected to surgical incisions. Two incisions were made on each pig, one being an experimental wound and the other a control wound. A bandage dressing was applied to cover each wound. The electro-pneumatic device was only attached to the bandage for the experimental wound. All of the devices were set to operate in pulsating mode at a frequency of approximately 0.28 Hz, and the air pressure value in the cuff was set to approximately 8.5 mmHg.

The experiment was conducted over four days following surgery^25^. Forty files of measurements were collected and stored, four files for each pig. A single file represented a day of air pressure monitoring in the cuff. All the acquired data were analysed using *Matlab software by MathWorks*, *Inc*. (see Supplementary Appendix C).

Table 2 summarizes the information about the testing period and times, listing the operation frequency and most significant values from the statistical analysis performed using the *MATLAB boxplot(X)* function, including the minimum, maximum and median values, whiskers and 25^th^ and 75^th^ percentile values. These results were obtained from an analysis of the signals using battery voltage values ranging from 12.3 to 11.5 V. Beyond the lower limit (i.e. 11.5 V), the value of battery voltage rapidly decreased (see Supplementary Fig. S2). Table 2 also shows the percentage values of the decreased pressure level while the device operated in pulsating mode, which ideally should have been approximately 70% of the pre-set value. The air pressure values were divided into three groups according to the value of calculated 75^th^ percentile. Pressure values of 12 to 21 mmHg were grouped in the first, values of 8 to 11 mmHg in the second and values of 4 to 7 mmHg in the third. The final column of Table 2 qualitatively indicates the success of the test, “YES” representing successful performance of the device, “NO” indicating unexpected results. The symbol “?” was assigned in cases when the value of the acquired air pressure was lower than the pre-set value. In tests number 9 and 10, the recorded value of the operation frequency was 0.11 Hz and did not correspond to the pre-set value of the operation frequency. All the graphs of trends of the acquired air pressure signals and the respective amplitudes of the Fourier transform are shown in the *Supplementary Material* (see Supplementary Figs S5–S8).

**Table 2:** Results of the electro-pneumatic device’s performance during the prospective study with animals.

	Total time (hh:mm:ss)	Oper. freq. (Hz)	Min. value (mmHg)	Max. value (mmHg)	Lower whisker (mmHg)	Upper whisker (mmHg)	Median value (mmHg)	25^th^ percentile value (mmHg)	75^th^ percentile value (mmHg)	Press. decr. (%)	Press. value groups	Was the test successful?
***TEST n*** **°** ***1***												
*first day*: 1_1	19:14:02	0.29	2.66	21.30	2.66	21.30	16.80	13.09	21.04	62	1^st^	NO
*second day*: 1_2	19:01:44	0.29	2.88	21.30	2.88	21.30	15.70	12.39	19.02	65	1^st^	NO
*third day*: 1_3	15:40:45	0.29	3.22	21.30	5.06	10.58	7.98	7.12	8.51	84	2^nd^	YES
*fourth day*: 1_4	19:32:56	0.29	2.71	21.30	4.91	10.63	7.99	7.05	8.48	83	2^nd^	YES
***TEST n*** **°** ***2***												
*first day*: 2_1	18:33:59	0.28	3.57	21.30	4.57	10.61	7.59	6.84	8.34	82	2^nd^	YES
*second day*: 2_2	18:51:30	0.28	3.73	21.30	4.75	10.13	7.43	6.76	8.11	83	2^nd^	YES
*third day*: 2_3	17:20:24	0.28	3.38	21.30	4.99	9.67	7.42	6.76	7.92	85	2^nd^	YES
*fourth day*: 2_4	18:20:33	0.28	3.60	21.30	4.98	9.42	7.33	6.65	7.76	86	2^nd^	YES
***TEST n*** **°** ***3***												
*first day*: 3_1	21:00:41	0.26	3.15	25.82	3.15	25.82	17.02	12.88	20.71	62	1^st^	NO
*second day*: 3_2	23:57:24	0.26	3.17	25.82	3.17	25.82	18.13	14.01	21.63	65	1^st^	NO
*third day*: 3_3	24:06:35	0.26	3.23	25.82	3.23	25.82	13.69	10.36	18.11	57	1^st^	NO
*fourth day*: 3_4	24:42:31	0.26	3.18	25.82	3.18	25.82	16.49	12.82	20.30	63	1^st^	NO
***TEST n*** **°** ***4***												
*first day*: 4_1	20:10:32	0.26	2.77	21.81	3.36	13.20	8.10	7.05	9.51	74	2^nd^	YES
*second day*: 4_2	23:57:04	0.26	2.78	21.81	3.98	10.63	7.34	6.48	8.14	80	2^nd^	YES
*third day*: 4_3	24:05:38	0.26	2.73	21.81	4.12	11.50	7.82	6.89	8.73	79	2^nd^	YES
*fourth day*: 4_4	24:45:43	0.26	2.73	21.81	4.21	10.59	7.45	6.60	8.20	81	2^nd^	YES
***TEST n*** **°** ***5***												
*first day*: 5_1	19:27:50	0.25	3.27	25.82	4.96	11.66	8.39	7.48	9.15	82	2^nd^	YES
*second day*: 5_2	—	—	—	—	—	—	—	—	—	—	—	—
*third day*: 5_3	23:54:35	0.25	3.36	25.82	4.88	11.54	8.38	7.37	9.04	82	2^nd^	YES
*fourth day*: 5_4	24:57:54	0.25	3.31	25.82	4.96	11.24	8.25	7.32	8.89	82	2^nd^	YES
***TEST n*** **°** ***6***												
*first day*: 6_1	18:47:10	0.26	3.48	25.82	3.48	25.82	15.76	11.23	20.51	55	1^st^	NO
*second day*: 6_2	16:57:14	0.26	3.40	25.82	3.40	25.82	15.39	10.28	21.15	49	1^st^	NO
*third day*: 6_3	23:58:44	0.26	3.21	25.82	4.49	11.13	7.80	6.98	8.64	81	2^nd^	YES
*fourth day*: 6_4	23:11:20	0.26	3.31	25.82	3.31	25.82	15.50	10.77	20.95	51	1^st^	NO
***TEST n*** **°** ***7***												
*first day*: 7_1	21:15:11	0.26	3.13	24.93	3.13	7.04	4.53	3.90	5.47	71	3^rd^	?
*second day*: 7_2	23:58:10	0.26	3.15	25.82	3.15	7.29	4.45	3.86	5.19	74	3^rd^	?
*third day*: 7_3	23:47:12	0.26	3.13	25.82	3.13	23.43	5.94	3.78	11.03	34	1^st^	NO
*fourth day*: 7_4	24:51:51	0.26	3.13	25.76	3.13	23.05	10.27	6.02	13.60	44	1^st^	NO
***TEST n*** **°** ***8***												
*first day*: 8_1	20:25:43	0.26	2.64	21.81	2.64	12.90	5.70	4.51	7.90	57	3^rd^	?
*second day*: 8_2	24:06:32	0.26	2.64	21.81	2.64	10.04	5.05	3.96	6.78	58	3^rd^	?
*third day*: 8_3	23:46:49	0.26	2.64	21.81	2.64	9.64	4.19	3.32	5.60	59	3^rd^	?
*fourth day*: 8_4	—	—	—	—	—	—	—	—	—	—	—	—
***TEST n*** **°** ***9***												
*first day*: 9_1	19:43:45	0.11	3.26	25.82	3.26	8.04	4.51	3.76	5.62	67	3^rd^	?
*second day*: 9_2	24:07:24	0.11	3.26	25.82	3.26	10.00	5.20	3.54	6.26	57	3^rd^	?
*third day*: 9_3	23:46:27	0.11	3.20	25.82	3.20	9.63	4.96	3.55	6.73	53	3^rd^	?
*fourth day*: 9_4	24:55:35	0.11	3.24	25.82	3.24	7.92	3.65	3.36	5.24	64	3^rd^	?
***TEST n*** **°** ***10***												
*first day*: 10_1	19:06:14	0.11	3.15	20.56	3.15	7.64	3.83	3.45	4.82	72	3^rd^	?
*second day*: 10_2	24:07:24	0.11	3.18	25.82	3.18	6.55	3.93	3.47	5.77	60	3^rd^	?
*third day*: 10_3	23:45:49	0.11	3.15	25.82	3.15	7.00	3.51	3.39	4.73	72	3^rd^	?
*fourth day*: 10_4	24:57:42	0.11	3.18	16.91	3.18	5.35	3.60	3.45	4.10	84	3^rd^	?

**Figure 3:**
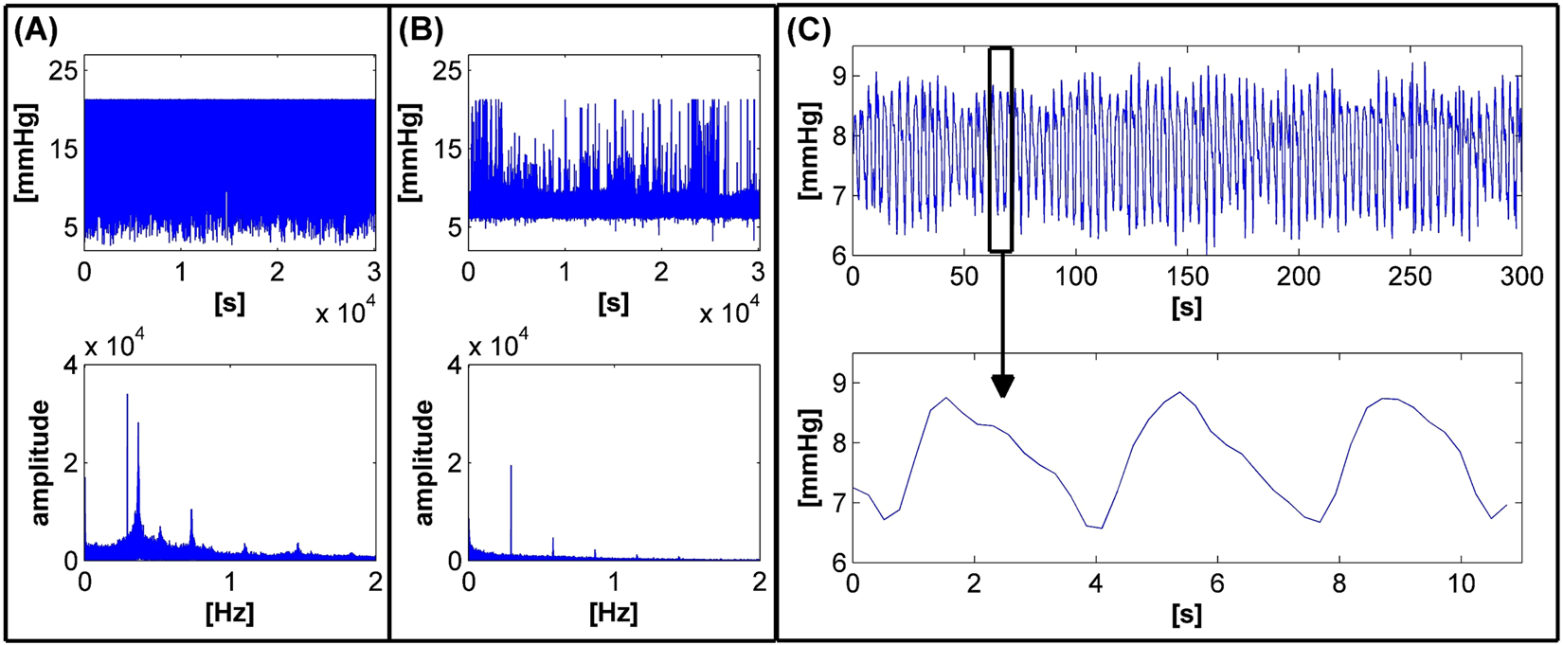
The trends of unexpected (**A**) and expected (**B**) acquired signals with relative Fourier transform amplitudes. Box (**C**) shows enlargements of the expected air pressure signal in the cuff.

Figure 3 shows the trends of unexpected (Fig. 3A) and expected (Fig. 3B) acquired signals. Figure 3A shows the measurements from the first day of test number 1, and Fig. 3B shows the air pressure monitored on the third day of the same test. The air pressure signal data are displayed in the battery voltage value range of 12.3 V to 11.5 V. Figure 3A,B also show the amplitudes of the Fourier transforms related to both tests. In order to analyse proper trends in air pressure, Fig. 3C shows two enlargements of the signal acquired on the third day of test number 1 in the battery voltage value range of 12.3 V to 11.5 V (Fig. 3B). The first enlargement shows ten minutes of the acquired signal, the second shows approximately ten seconds of the test.

The signal displayed in Fig. 3A was identified as an incorrect trend, and conversely the trend displayed in Fig. 3B was designated a correct air pressure signal.

Figure 3A shows the air pressure signal saturated to the upper limit. Its reason was a problem to do with hose occlusion. When the hoses between the cuff and the electronic part of the system are occluded, air is not distributed in the cuff and causes the air pressure level to increase at the opposite side of the system. After a compression cycle, the high air pressure level measured by the transducer thus leads to a release of the valve, which drains the air from the system quickly, and the process starts again. Several causes could have likely occluded the hoses, for example, poor packing into the waterproof bag, or the animals perhaps pressed the hoses against the thin edges of the bandage, belt or bag clips or entangled them somehow.

Referring to Fig. 3B, we could evaluate the signal as correct, since the values of the air pressure were in the range of the pre-set values. Random peaks were observed while measuring the signal, however. Generally, the animal’s normal movement caused these peaks, which usually reached the saturation limit through the high gain of the amplifier.

Figure 3C represents a proper trend in the air pressure signal in the cuff. The enlargement in Fig. 3C clearly displays the behaviour of the device as it operated in pulsating mode. The operating frequency of the device was approximately 0.28 Hz, and the signal gradually decreased. The signal did not decrease down to the ideal value (i.e. 70%) of the pre-set value, but to 80%. This percentage value (i.e. the decreased level of air pressure in the cuff of 80%), was approximately the same in all the tests identified as correct.

In the tests marked with the symbol “?” in the final column of Table 2, human error may have been involved while setting the device parameters before beginning the test. Another possible reason for these results could have been a malfunction in the device caused by incorrect positioning of the instrument during testing. To help eliminate these possible issues in future testing, users will be given more training.

### Histological analysis

On the fifth day post-surgery, twenty specimens of skin tissue were collected to proceed with the histological analysis, ten being experimental wound specimens and ten control specimens. Each specimen was analysed according to the twelve healing parameters mentioned in the methods section.

Table S2 (see Supplementary Material) shows the values assigned to all the mentioned parameters. The columns marked “*X*_*E*_” refer to the values assigned to the experimental specimens, while the columns marked “*X*_*C*_” refer to the control specimens. The obtained data were paired because the values of the experimental and control wound specimens for each pig were compared. A Wilcoxon signed-rank test (α = 0.05) revealed a statistically significant difference (*p*_*b*.*m*.*d*._ = 0.021; see Supplementary Table S2) only in the *b*. *m*. *d*. histological parameter.

**Figure 4:**
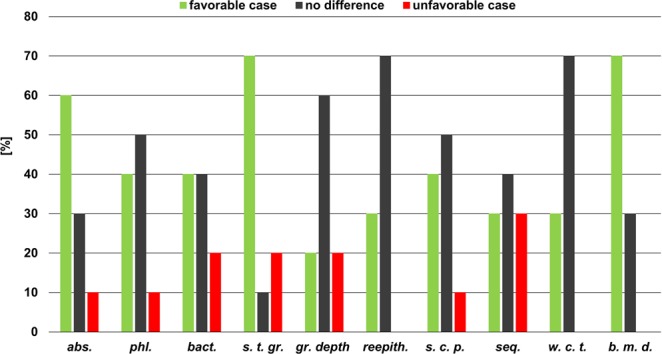
Results of the descriptive statistic performed to discover favourable (green), no difference (grey) and unfavourable (red) cases of wound healing in each analysed histological parameter.

For the descriptive statistic to deepen the analysis of the histological study, Fig. 4 summarizes the results of the contingency tables used to process the assigned discrete values (see Supplementary Tables S3–S12), except for the *cav*. *diam*., and *seq*. *diam*. parameters, which were measured in millimetres.

No unfavourable cases were observed for parameters *b*. *m*. *d*., *w*. *c*. *t*. and *reepith*. However, the results for *w*. *c*. *t*. and *reepith*. showed that most cases (i.e. 70%) had no differences between the two compared specimens. By contrast, the *b*. *m*. *d*. and *s*. *t*. *gr*. parameters both obtained the highest number of favourable cases (70%). The s. *t*. *gr*. *parameter*, however, also demonstrated a percentage of unfavourable cases (20%). The *abs*. parameter obtained a result of 60% of favourable cases, while the *phl*., *bact*. and *s*. *c*. *p*. parameters showed a percentage of favourable cases of less than 50%. The *gr*. *depth* and *seq*. parameters showed the worst results, showing percentages of favourable cases equal to the percentages of unfavourable cases. A summary of the results in Fig. 4 indicates that favourable cases and cases in which no difference was found were equivalent, while unfavourable cases were a restricted minority. The results for the *cav*. *diam*. and *seq*. *diam*. parameters show in only 10% of cases that the measured difference was higher than or equal to the threshold value stated by the physicians (i.e. 5 mm). According to the pathologist’s assessment, the evidence for the healing process using the electro-pneumatic device was questionable, exhibiting a high variability in results because of the low number of samples considered in the prospective study with animals^25^.

Nevertheless, in order to statistically analyse the results for each single test (discrete values listed in Supplementary Table S15, *n* = 10, paired data), a Wilcoxon signed-rank test (α = 0.05) was conducted to reveal the differences between the experimental and control wounds in all the animals. The results show statistically significant differences in the 5^th^ (*p*_5_ = 0.031), 9^th^ (*p*_9_ = 0.021) and 10^th^ (*p*_10_ = 0.013) tests, which represents 30% of the performed tests.

**Figure 5:**
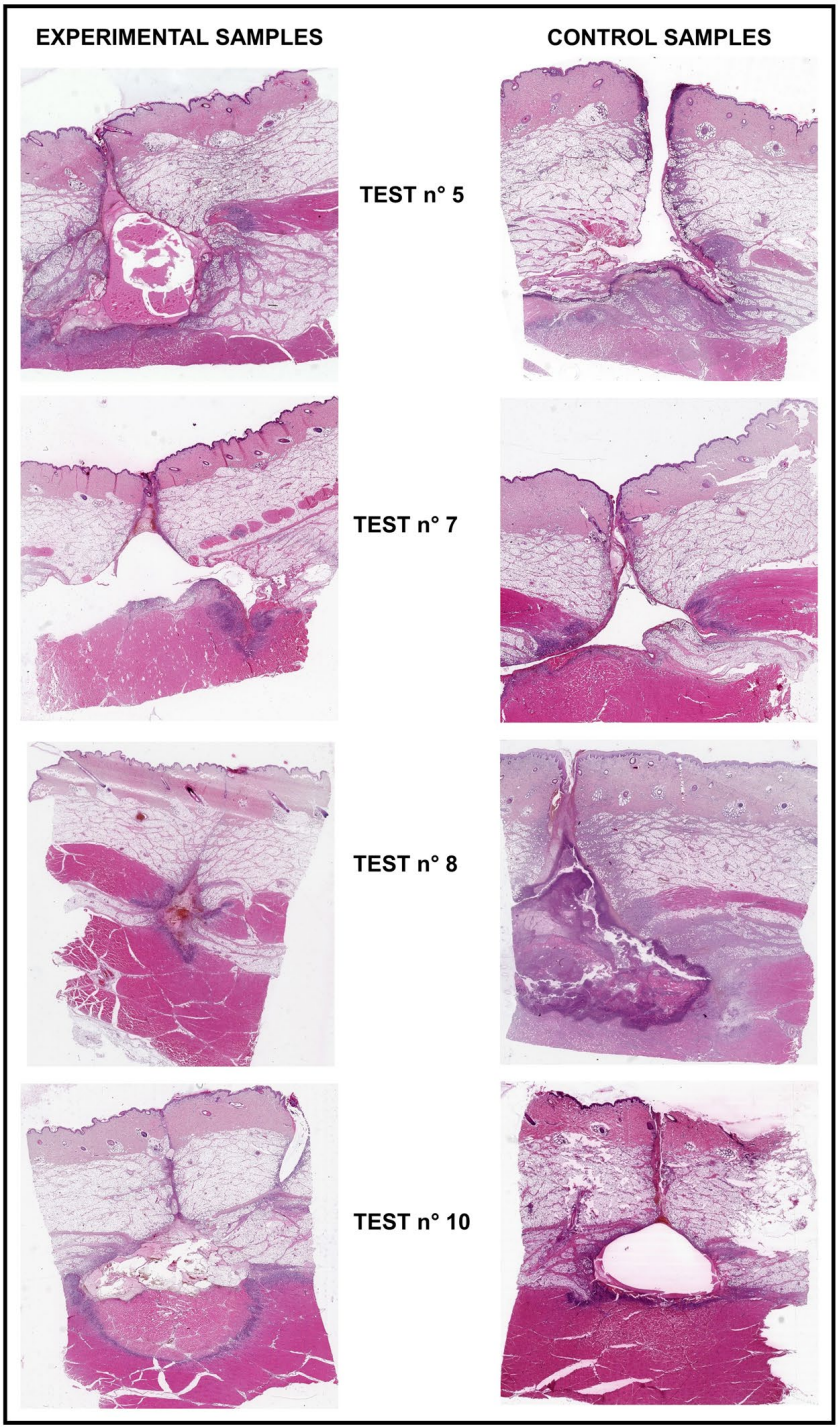
Macrophotographs of the wound specimens treated with the electro-pneumatic device (i.e. experimental samples) at left side, and the non-treated specimens (i.e. control samples) at right.

Figure 5 shows macrophotographs of the wound specimens for tests number 5, 7, 8 and 10. The experimental samples are shown at left, the control samples at right. A solution of haematoxylin and eosin was used to stain all samples. Figure 5 verifies the high variability of results shown in Fig. 4. Indeed, the experimental wounds in tests 5 and 10 indicate a greater benefit than in the corresponding control wounds. It is clearly visible in test 7 that healing in the control sample was better than in the experimental sample. Tests 5, 8 and 10 (Fig. 5) show an almost completely healed sinus in the experimental samples with over-epithelized surfaces, especially in test number 8, and the surrounding area also indicates eosinophilic reactions in the muscle tissue. The experimental specimens also have a large area of basophilic decomposition and granulation. The sinus tract in the control wound specimens did not always close and had sequesters of necrotic tissue and bacteria.

## Discussion

In the current scientific literature, the most-used therapies described to accelerate wound healing are negative pressure therapy, electrical stimulation and hyperbaric oxygen therapy^26^. No evidence of data indicates one specific therapy prevailing over any other. Compression therapy could therefore potentially be widely adopted in clinical practice, as reported by Partsch & Mortimer^27^ and Dolibog *et al*.^28^.

On the commercial market, devices such as those in this experiment are categorized as intermittent pneumatic compression (IPC) devices. However, these instruments are mostly used on the lower limbs and provide high values of air pressure to the skin. For example, physicians used the Kendall SCD™ Compression System^29^ to prevent venous thromboembolism after a primary total hip arthroplasty^30^. This device can provide a compression of 130 mmHg in five seconds. IPC devices are frequently used to manage lymphedema and nonhealing venous leg ulcers. Zaleska *et al*. used Bio Compression Systems^31^ products to apply pneumatic compression in order to obtain efficient tissue fluid flow and sufficient lymph flow in limbs^32^. Mayrovitz compared two types of lymphedema therapy devices: Lympha Press^33^, a traditional compression pump, and the Flexitouch System^34^, an alternative compression pattern device. The pressure values generated from these devices were considerably different. The author stated that the proper instrument for therapy must be selected according to the patient’s needs^35^. For venous ulcer healing, Nikolovska *et al*. analysed treatment time by applying rapid and slow ICP using the Green Press 12 device, Iskra Merdical^36^. In their results, the authors concluded that rapid ICP is more effective than slow ICP during the healing of venous ulcers^37^. IPC devices are also used to enhance neurovascular ingrowth and tissue proliferation during connective tissue healing. *Dahl et al*. proposed a study with animals in which IPC treatment accelerated the repair process during the healing of Achilles tendon rupture^38^. In this study, the CRYO/CUFF® device^39^ was used to provide automated cold and compression therapy and thereby reduce postoperative swelling.

The properties of these commercial devices are summarized in Table 3. Of these devices, the Flexitouch System is the only capable of providing treatment in the neck area. Mayrovitz *et al*. conducted a prospective study to assess the device’s usability and patient comfort during therapy for head and neck lymphedema. The results showed that the system brought significant benefits to patients, self-managing the lymphedema in the head and neck area and thereby improving the patient’s quality of life^40^.

**Table 3:** Comparison of the properties of commercial IPC devices to the device proposed in this paper.

Device	Garments	Set pressure [mmHg]	Compression cycle	Controller sizes	Ref.
*Kendall SCD EXPRESS*	Up to two garments can be attached to the controller: leg sleeve(s) and/or foot cuff(s).	Leg Sleeves: 45 mmHg Foot Cuffs: 130 mmHg	Leg Sleeves: 11 s compression Foot Cuffs: 5 s compression Decompression time based on Vascular Refill Detection measurement	Height: 15.8 cm Width: 17.8 cm Depth: 11.4 cm	^29^
*Bio Compression Systems*, *Inc*.	Four or eight discrete inflatable chambers over the affected limb or torso.	30–125 mmHg.	Inflation: 54 s Deflation: 6.5 s Cycle time: 6.5 s/chamber	Height: 11.4 cm Width: 29.9 cm Depth: 19.7 cm	^31^
*Lympha Press Optimal®*	Twelve to twenty-four chambers, including leg and arm garments, Lympha Pants™, ComfySleeve™ and Lympha Jacket™.	20–90 mmHg.	Inflation: 26 s Deflation: 4 s	Height: 15.0 cm Width: 32.0 cm Depth: 38.5 cm	^33^
*Flexitouch® Plus System*	Up to thirty-two chambers in the garments. Lower and upper extremity garments, head garments and vests for the head and neck.	Pressure values based on the physiological movement of lymph fluid through system. (20–40 mmHg^34^)	Five available cycles	Height: 20.3 cm Width: 25.4 cm Depth: 20.3 cm	^34^
*Green Press 12*	Different garments for the leg, foot, waist, hips and arm.	0–165 mmHg.	Pressure duration: 0–90 s	Height: 50.0 cm Width: 41.0 cm Depth: 22.0 cm	^36^
*Our device*	A garment can be attached to the controller.	3–11 mmHg	Pressure duration: 3.3–9 s	Height: 3.3 cm Width: 14.7 cm Depth: 8.0 cm	This paper

The device proposed in this paper has small controllers, can provide compression therapy in the head and neck area and has the advantage of generating extremely low-pressure values in the range of approximately 3 to 11 mmHg. It can also reduce air pressure in the cuff to 70–80% of its pre-set value with an adjustable operating frequency between 0.1 and 0.3 Hz. Its disadvantage is that even minute, unwanted perturbations can modify the pre-set air pressure value. The maximum values, for example, 19.68, 21.30, 21.81 and 25.82 mmHg in Tables 1 and 2, indicate that the cuff experienced external perturbations during the experiments. External perturbations could be any touch with the fingers or any body movement that compresses the cuff. To overcome this problem, either patients need to remain completely motionless or scientists could find a design solution that guarantees proper protection of the cuff.

Another limitation is the power consumption of its active parts (i.e. the electro-valve and air compressor). This large power consumption led to the use of a heavy and bulky battery, which limited the portability of the device. The main use of the proposed device is therefore with patients at home or in hospital confined to bed.

During the experiment in the prospective study with animals, an operating frequency of approximately 0.28 Hz and air pressure value of approximately 8.5 mmHg were set. A Wilcoxon signed-rank test conducted to reveal the differences between the experimental and control wounds in all of the analysed histological parameters (see Supplementary Table S2) showed a significant difference only in the *b*. *m*. *d*. parameter (*n* = 10; *p*_*b*.*m*.*d*._ = 0.021). To explore this result, a further descriptive statistic was performed to compare the discrete values assigned to the histological parameters. The histograms in Fig. 4 show the percentages of favourable, no difference and unfavourable cases observed after treatment. Although Fig. 4 indicates a predominance of favourable and no-difference cases over unfavourable cases, a high variability in the results can be seen, mainly because of the low number of samples analysed in the experiment (*n* = 10).

Another Wilcoxon signed-rank test was performed to statistically analyse the differences between the experimental and control wounds in all the treated animals (see Supplementary Table S15) and revealed a significant difference in tests 5, 9 and 10. This result indicates that only the 30% of the tests obtained a physiological benefit by using the electro-pneumatic device.

It should be noted that tests number 9 and 10, which obtained the best statistical results (*n* = 10; *p*_9_ = 0.021 and *p*_10_ = 0.013), were conducted using different values in the device: air pressure was set to 4–7 mmHg at a pulsating frequency of 0.11 Hz (Table 2). Combined with the low number of samples, this further increased the variability of the results, and from a medical point of view, the evidence for improved healing process using the electro-pneumatic device is questionable^25^. The variability in the results could also be attributed to the animals’ movements as they lay on the table during the experiment.

Figure 5 confirms the variability in the results. The experimental wounds in tests 5 and 10 healed better than the corresponding control wounds. Conversely, the control wound in test number 7 healed better than the experimental wound in terms of residual cavity.

A comparison of the data in Table 2 with the statistical results for all the treated animals shows that the best healing process (i.e. tests 9 and 10, where *n* = 10; *p*_9_ = 0.021 and *p*_10_ = 0.013) occurred when the values of the device were at their lowest settings (i.e. an air pressure of 4–7 mmHg and pulsating frequency of 0.11 Hz). It may mean that this change in pressure amplitude and operation frequency values led to a greater benefit than those obtained in the other tests. It can be concluded that the electro-pneumatic device potentially improves the wound healing process in the neck area if the pre-set value of air pressure does not exceed 8 mmHg. In future clinical tests, the pre-set air pressure value will be maintained at a lower pressure value than in this study.

The proposed device may also help the flow of lymph into capillaries to reduce postoperative swelling. Indeed, lymphatic capillary pressure is in the range of 4 to 8 mmHg, as reported by Spiegel *et al*.^41^ and Zaugg-Vesti *et al*.^42^ In terms of compression of skin tissue, the performance of the device makes it suitable for IPC therapy. By changing the value of some passive components in the electronic circuit, such as the resistors and capacitors, it could also generate higher values of air pressure in the cuff and thus be adapted to different patient needs. As a possible improvement to the device in future applications, its pulse frequency could be synchronized with the patient’s blood flow oscillation frequency.

Research in wearable technologies for medical applications has found new solutions for the wound healing process. One such wearable device, for example, is small, very light and thin and comprises a control module and wire antenna for pulsed radio frequency therapy of recalcitrant ulcers^43^. The non-invasive effects of non-thermal plasma in pressure ulcer treatment also accelerate wound healing^44^, and a miniaturized instrument for sutures could promote the healing process by delivering heat and electrical stimulation to the wound^45–47^. Scientists all over the world are studying healing solutions based on the delivery of proteins and stem cells^48–53^, which could be incorporated into biocompatible and biodegradable polymers for medical applications^54,55^. These solutions take the form of simple patches that allow patients to wear the healing systems with ease and provide a significant benefit to personal well-being. While examining the possibility of producing intermittent mechanical pulses on the skin through small patches, electro-active polymer materials could be a promising solution, as they possess the ability to generate forces when excited by differences of electric potential^56^. However, they require high driving voltages to operate, and their common use today is only in monitoring mechanical pulses rather than care therapies^57,58^.

## Conclusions

We successfully developed an electro-pneumatic device capable of providing soft mechanical pressure on human tissue. The mechanical pressure can be either continuous or pulsating. The range of air pressure values in the cuff is 3 to 11 mmHg and the range of operation frequency for the pulsating pressure mode is 0.1 to 0.3 Hz. The results of the laboratory measurements showed correct functioning of the device in the range of values mentioned above, both in pressure and operating frequency. Because of the low sample number examined during the prospective study with animals (*n* = 10), the results demonstrated a high variability. Out of twelve histological parameters analysed by the pathologist, only the basophilic muscle decomposition parameter indicated a statistically significant difference between experimental and control wounds. Out of ten animals treated with the electro-pneumatic device, only three (tests 5, 9 and 10) indicated a statistically significant therapeutic benefit between the experimental and control wounds. Out of these three, tests 9 and 10 were conducted with an accidental setting of the lowest air pressure values in the cuff. Although human error was assumed in setting the air pressure value, future trials on humans will be performed with pressure values of less than 8 mmHg. In order to strictly verify whether the proposed device genuinely improves blood perfusion in areas surrounding a wound, instrumentation should be added to measure the values of blood circulation into capillaries and tissue oxygenation, and other vein tests should be conducted.

## Supplementary information

Supplementary Material

Dataset 1
