# Supplementary material for: Evaluation of an Electro-Pneumatic Device for Artificial Capillary Pulse Generation used in a Prospective Study in Animals for Surgical Neck Wound Healing
Source: Sci Rep. 2019 Jul 8;9:9837. doi: 10.1038/s41598-019-46397-0 (PMC6614409; doi:10.1038/s41598-019-46397-0)
Supplement: Supplementary file 1 — Supplementary Material [file 41598_2019_46397_MOESM1_ESM.pdf]

Supplementary Material – Evaluation of an Electro-Pneumatic Device for Artificial Capillary Pulse Generation used in a Prospective Study in Animals for Surgical Neck Wound Healing

J. Foltyn[a,1], A. Proto[b,1,*], D. Oczka[b], R. Halfar[b], T. Klinkovsky[b], L. Skoloudik[c], M. Cerny[c], V. Chrobok[c], A. Ryska[d], V. Radochova[e], M. Litschmannova[f], M. Penhaker[b] & J. Mejzlik[c]

[a] Special Medical Technology Co., Ltd., Prague, Czech Republic.

[b] Department of Cybernetics and Biomedical Engineering, VSB - Technical University of Ostrava, Czech Republic.

[c] Department of Otorhinolaryngology and Head and Neck Surgery, University Hospital Hradec Kralove, Charles University, Faculty of Medicine in Hradec Kralove, Czech Republic.

[d] Fingerland's institute of Pathology, University Hospital Hradec Kralove, Charles University, Faculty of Medicine in Hradec Kralove, Czech Republic.

[e] Vivarium, Faculty of Military Health Sciences, University of Defence, Brno, Czech Republic.

[f] Department of Applied Mathematics, VSB - Technical University of Ostrava, Czech Republic.

[*]Corresponding author. Email: antonino.proto@vsb.cz

[1] These authors contributed equally to this work

In **Figure S1**, the dashed oval circle (red colour) indicates attenuation of the amplitude of blood pressure oscillation as blood passes from the arteries to capillaries[1].

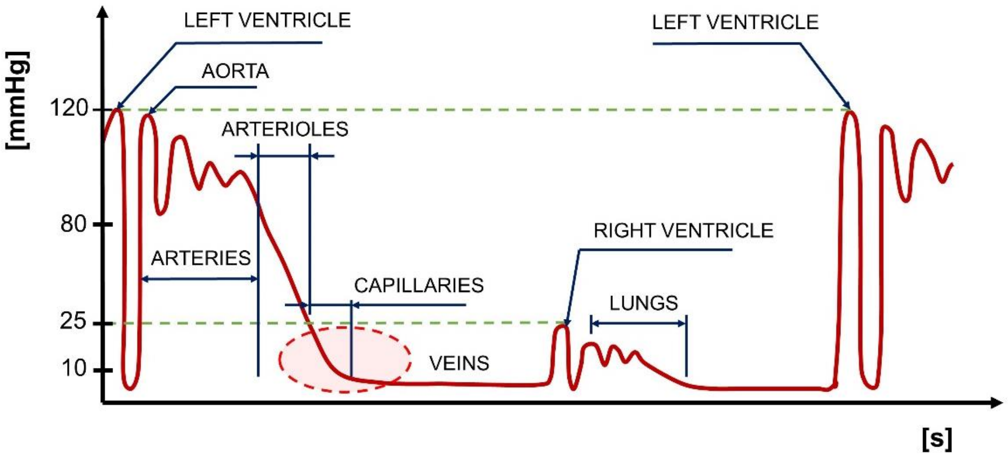

Figure S1. *Attenuation of the amplitude of blood pressure oscillation as blood passes from arteries to capillaries.*

Table S1 shows the electrical specifications in terms of maximum voltage supply and peak currents, the recommended voltage supply value and the value of current consumption when the set air-pressure in the cuff is approximately 10 mmHg.

Table S1. *Electrical specification of the electro-pneumatic device.*

Parameter	*Value* MIN	TYP	MAX	*Unit*
Absolute maximum voltage supply	5.5	-	15	V
Maximumn peak current	-	-	600	mA
Recommended voltage supply	11	12	15	V
Average current consumption (pressure value set at 10 mmHg)	-	150	-	mA

Figure S2 shows the battery discharge curve and corresponding voltage values. The LED light changes colour from green to red.

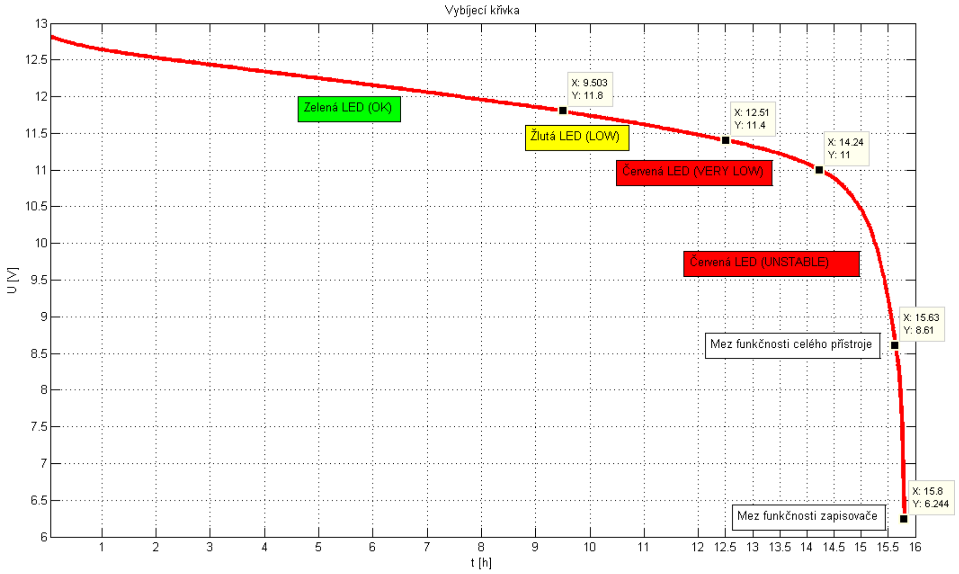

Figure S2. *Battery discharge curve.*

Figure S3 shows the response of the calibration curve in each developed device. All of the curves have a linear tendency, with an offset value that can be ignored.

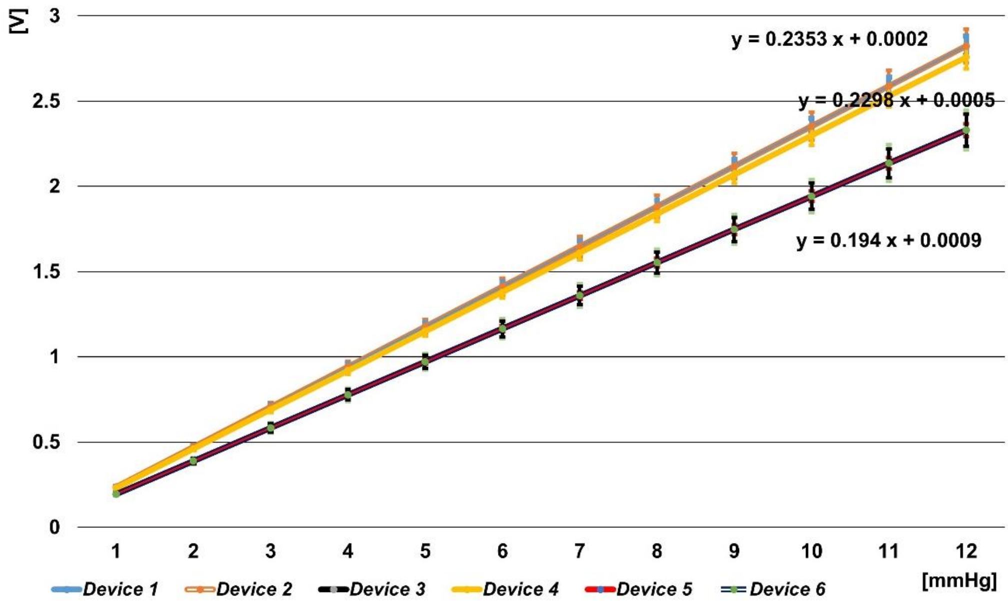

Figure S3. *Response of the calibration curve in each developed device. Error bars represent standard deviations.*

Appendix A

Below is the procedure to calculate the values of sensitivity the resolution parameters.
It is important to note that the response of calibration curve has a linear trend and a negligible offset in each of the six developed devices (**Fig. S3**).

The sensitivity value (S) is calculated as follows[2]: $S = \frac{\Delta V_{out}}{\Delta P_{in}}$.

Since ΔP_{in} = 1 mmHg, sensitivity results in approximately 0.22 V/mmHg. It is the calculated mean value of all values measured in each device.

The resolution value (R) can be calculated as follows[2]:

$$R = \lim_{\Delta V_{out} \to V_{noise}} \frac{\Delta V_{out}}{S} = \frac{V_{noise}}{S}$$

The values of V_{noise} are given by the sum of all noise values related to the components used to measure the signal (**Fig. Appendix A**). The signal crosses ten resistors and two operational amplifiers. In the ten resistors, the root mean square noise voltage is the result of calculating the thermal noise[3], also called Johnson noise, at a system bandwidth of 10 Hz, while the value of noise of the two operational amplifiers is given by the datasheet. As result, the voltage noise of the analogue circuit for data acquisition is approximately 10 µV.

In the digital circuit, the signal sampling introduces additional noise (i.e. quantization noise). The 10-bit A/D converter divides the reference voltage (i.e. 5 V) into 1023 sub-intervals, each being approximately 5 mV. This is the A/D converter resolution.

In practice, the resolution value (R_{real}) is obtained from values approximately five times greater than the voltage noise level[4]. Therefore, the resulting resolution value is approximately 0.11 mmHg.

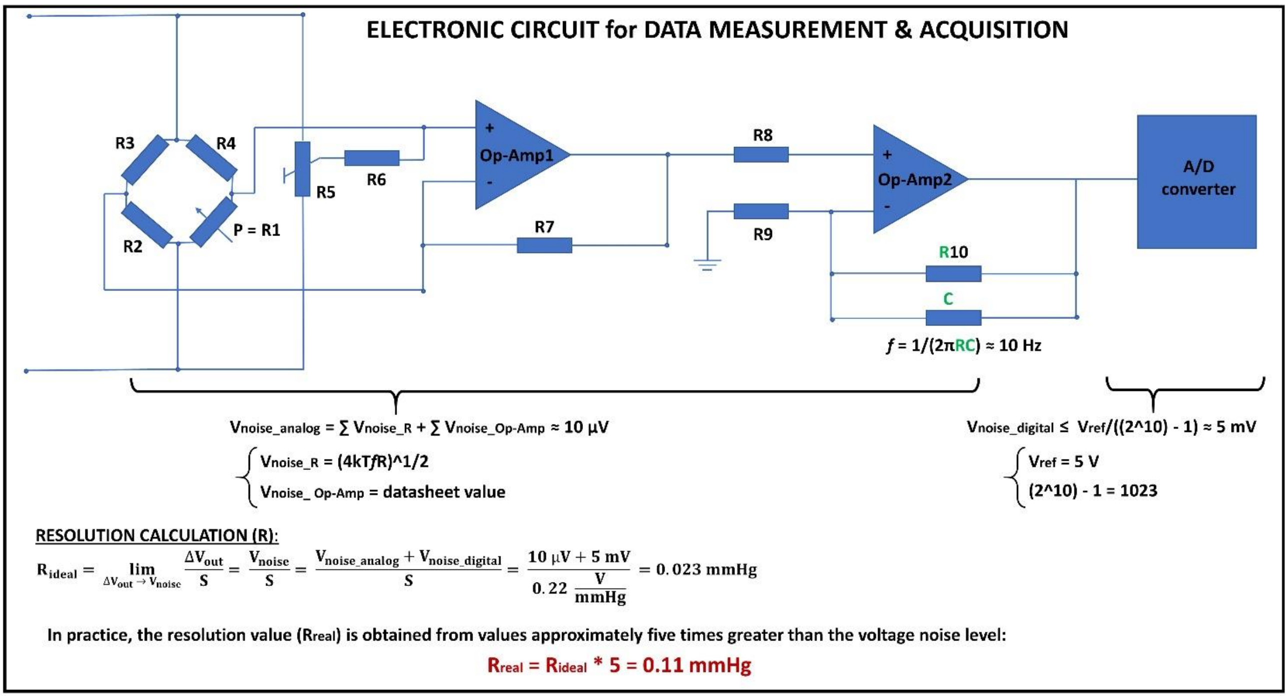

Figure Appendix A. *Electronic circuit for data measurement and acquisition. It explains the method used to calculate resolution.*

Fig. S4 shows the relationship between the slider position of potentiometers and pre-set values of operating frequency (**Fig. S4a**) and air pressure (**Fig. S4b**).

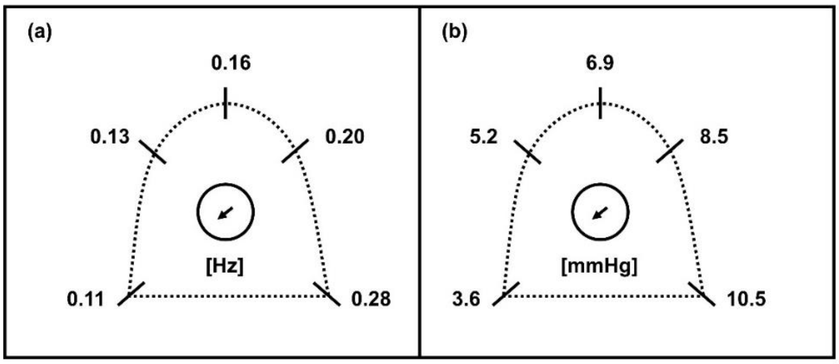

Figure S4. *Illustation of the slider position of the potentiometer relative to the measured value:* ***(a)*** *frequency,* ***(b)*** *air pressure.*

Appendix B

The results presented in **Table 1** and **Figure 2** were obtained by the following procedure:

A. **Table 1** results.
   a. Open *Matlab software, MathWorks, Inc*..
   b. Set the access path to the folder directory *Matlab_Codes*. All data are stored here.
   c. Select the *TAB_1* folder.
   d. For ***Continuous Mode*** results, open the *Continuous_Mode* folder.
      i. Load the *tab_1_continuous_mode.mat* workspace.
      ii. Open the *Pressure_Values_Graphs_continuous_mode.txt* file in the editor and copy all its content.
      iii. Paste it into the Command Window and run to visualize the graphical results.
      iv. In the figures that are opened, use the pointer to select points of interest.
      v. On the Command Window, use *clear;clc*; commands to clean the workspace and Command Window.
   e. For ***Press. Value n°1*** results, open the *Press_Value_1* folder.
      i. Load the *tab_1_press_value_1.mat* workspace.
      ii. Open the *Fourier_Transforms_press_value_1.txt* file in the Editor, and copy all its content.
      iii. Paste it into the Command Window and run to visualize the graphical results.
      iv. Open the *Pressure_Values_Graphs_press_value_1.txt* file in the editor and copy all its content.
      v. Paste it into the Command Window and run to visualize the graphical results.
      vi. In the figures that are opened, use the pointer to select points of interest.
      vii. On the Command Window, use *clear;clc;* commands to clean the workspace and Command Window.
   f. For ***Press. Value n°2*** results, open the *Press_Value_2* folder.
      i. Load the *tab_1_press_value_2.mat* workspace.
      ii. Open the *Fourier_Transforms_press_value_2.txt file* in the Editor, and copy all its content.
      iii. Paste it into the Command Window and run to visualize the graphical results.
      iv. Open the *Pressure_Values_Graphs_press_value_2.txt* file in the Editor, and copy all its content.
      v. Paste it into the Command Window and run to visualize the graphical results.
      vi. In the figures that are opened, use the pointer to select points of interest.
      vii. On the Command Window, use *clear;clc;* commands to clean the workspace and Command Window.
   g. For ***Press. Value n°3*** results, open the *Press_Value_3* folder.

i. Load the *tab_1_press_value_3.mat* workspace.
ii. Open the *Fourier_Transforms_press_value_3.txt file* in the Editor and copy all its content.
iii. Paste it into the Command Window and run to visualize the graphical results.
iv. Open the *Pressure_Values_Graphs_press_value_2.txt* file in the Editor and copy all its content.
v. Paste it into the Command Window and run to visualize the graphical results.
vi. In the figures that are opened, use the pointer to select points of interest.
vii. On the Command Window, use *clear;clc;* commands to clean the workspace and Command Window.

h. For ***Press. Value n°4*** results, open the *Press_Value_4* folder.
   i. Load the *tab_1_press_value_4.mat* workspace.
   ii. Open the *Fourier_Transforms_press_value_4.txt* file in the Editor, and copy all its content.
   iii. Paste it into the Command Window and run to visualize the graphical results.
   iv. Open the *Pressure_Values_Graphs_press_value_4.txt* file in the Editor, and copy all its content.
   v. Paste it into the Command Window and run to visualize the graphical results.
   vi. In the figures that are opened, use the pointer to select points of interest.
   vii. On the Command Window, use *clear;clc;* commands to clean the workspace and Command Window.
i. For ***Press. Value n°5*** results, open the *Press_Value_5* folder.
   i. Load the *tab_1_press_value_5.mat* workspace.
   ii. Open the *Fourier_Transforms_press_value_5.txt* file in the Editor, and copy all its content.
   iii. Paste it into the Command Window and run to visualize the graphical results.
   iv. Open the *Pressure_Values_Graphs_press_value_5.txt* file in the Editor, and copy all its content.
   v. Paste it into the Command Window and run to visualize the graphical results.
   vi. In the figures that are opened, use the pointer to select points of interest.
   vii. On the Command Window, use *clear;clc;* commands to clean the workspace and Command Window.

B. **Figure 2** results.
   a. Open *Matlab software by MathWorks, Inc*.
   b. Set the access path to the folder directory *Matlab_Codes*. All data are stored here.
   c. Select the *Fig_2* folder.
   d. Select the *Visualization_500_s* folder.
   e. Load the *matlab_final_filtering.mat* workspace.
   f. Open the *Visualize_500_s.txt* file in the editor and copy all its content.
   g. Paste it into the Command Window and run to visualize the graphical results.
   h. On the Command Window, use *clear;clc;* commands to clean the workspace and Command Window.

The *Fig_2* folder has numerous sub-folders (*Fix_freq_X_filtering*) that include all *.txt* files showing the steps to filter the signals. In this case, it needs to be opened the following workspace: *matlab_pulsating_mode_together.mat*.

The following steps were used to filter the signals. The same procedure was used for all the acquired signals.

1. Visualization of the measured signal over the time. Since the device stores data in milli-seconds, the signal was re-interpolated to visualize it over seconds.

2. Visualization of the Fourier Transform of the signals to select the signal main frequency component.
3. Creation of the filter coefficients according to the selected main frequency component.
4. Generation of the filtered signal.
5. Visualization of the filtered signal over the measured signal.

Figures S5–S6 show the trends of the acquired air-pressure signals. **Figure S5** refers to the signals for tests 1 to 5, and **Figure S6** refers to the signals for tests 6 to 10.

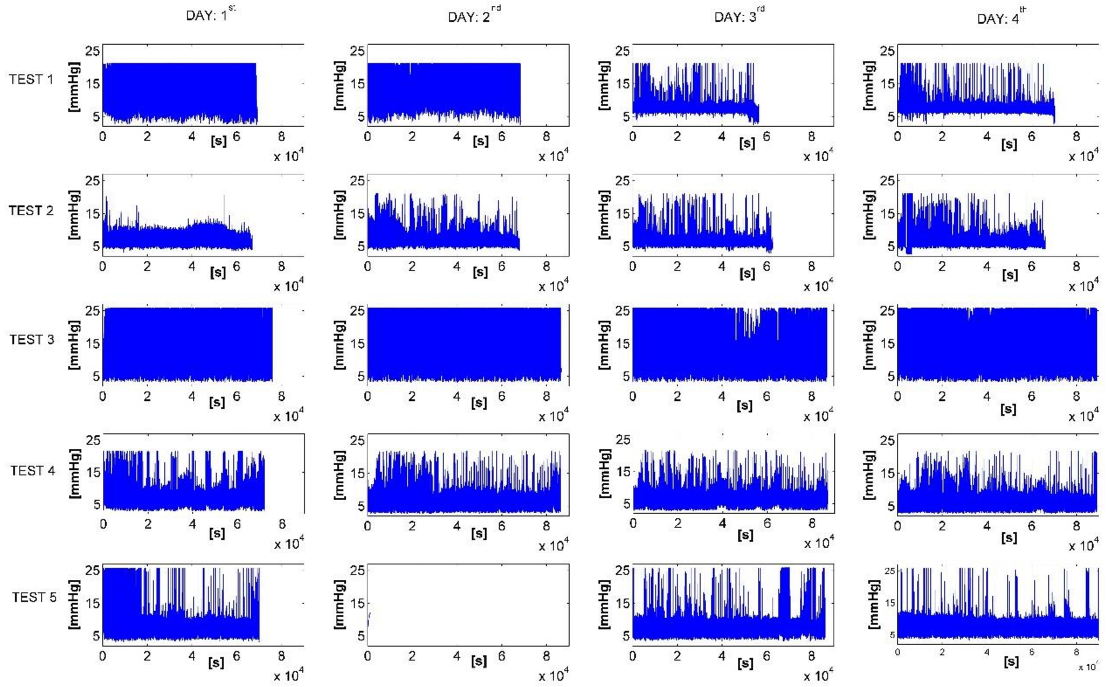

Figure S5. *Air-pressure signals for tests 1 to 5.*

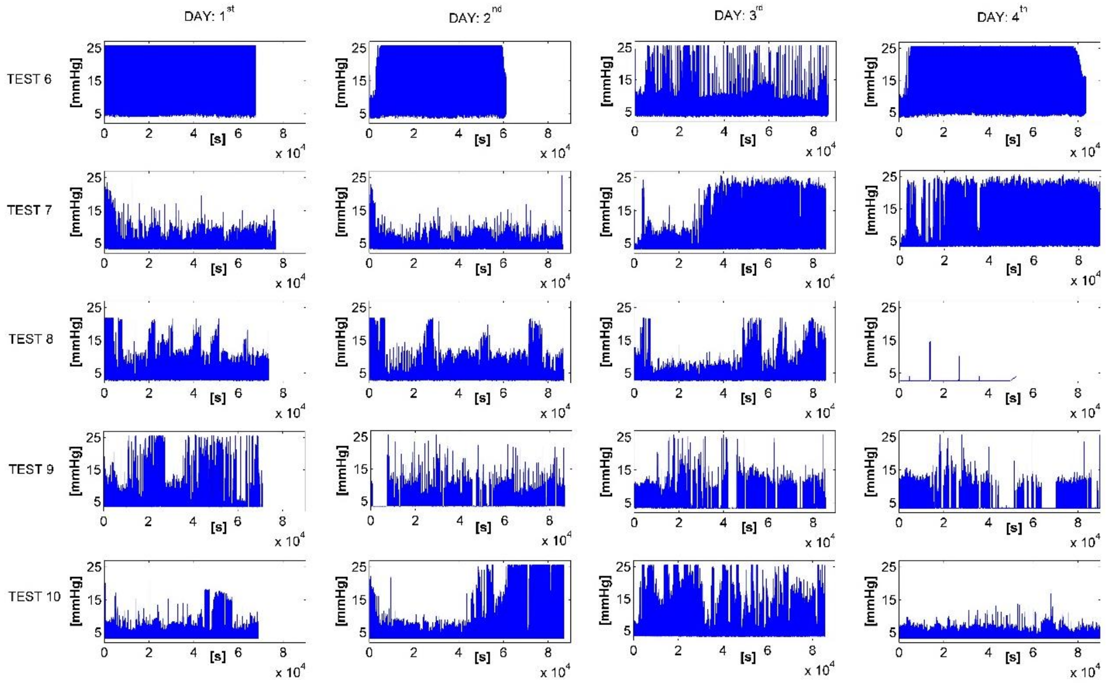

Figure S6. *Air-pressure signals for tests 6 to 10.*

Figures S7–S8 show the Fourier transform amplitudes of the acquired air-pressure signals. **Figure S7** refers to the Fourier transform amplitudes for tests 1 to 5, and **Figure S8** refers to the Fourier transform amplitudes for tests 6 to 10.

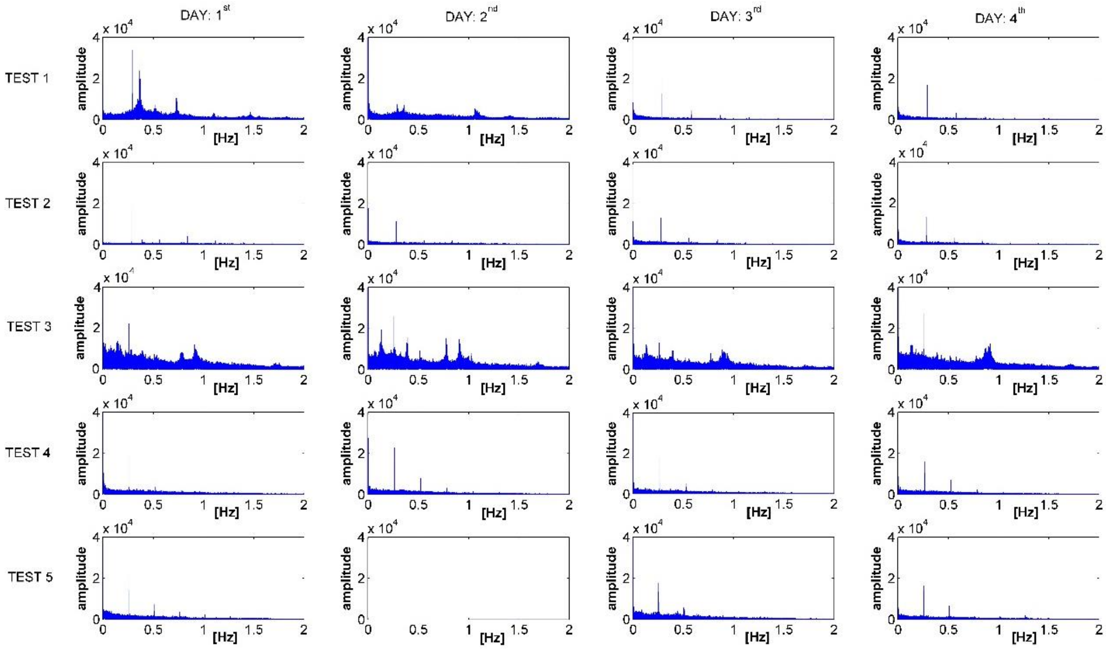

Figure S7. *Amplitudes of the Fourier transforms for tests 1 to 5.*

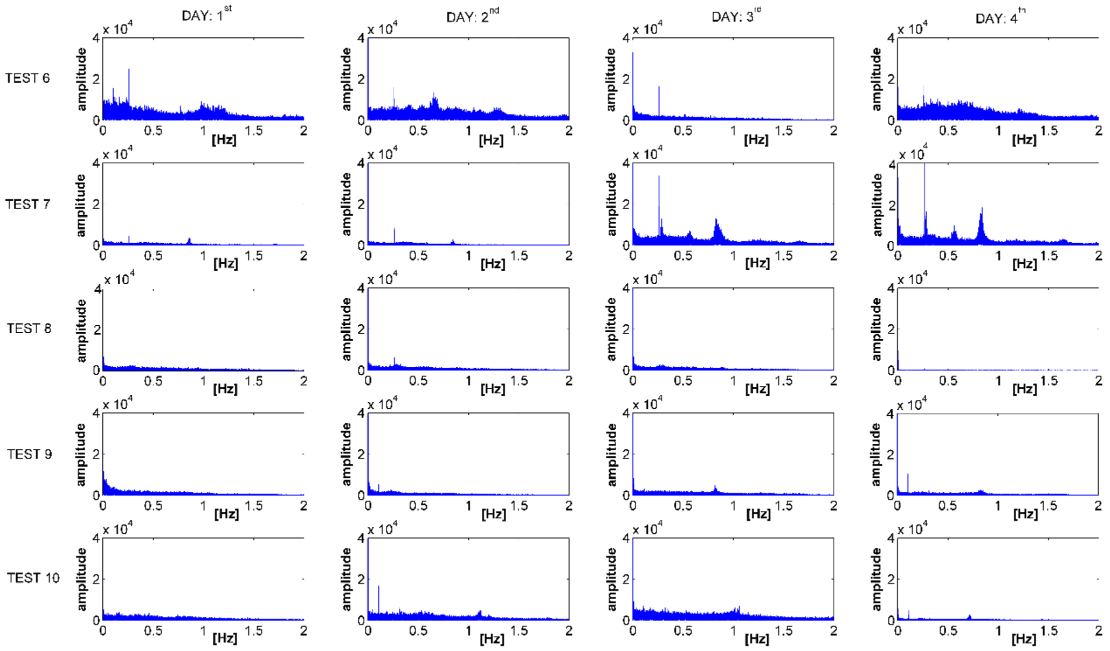

Figure S8. *Amplitudes of the Fourier transforms for tests 6 to 10.*

Appendix C

The following steps were used to obtain the results presented in **Table 2**.

A. Open *Matlab software by MathWorks, Inc*.
B. Set the access path to the folder directory *Matlab_Codes*. All data are stored here.
C. Select the *TAB_2* folder.

D. Load the *matlab_micro_perf.mat* workspace.
E. Open the *Micro_Perfusion_Graph.txt* file in the editor and copy all its content.
F. Paste it into the Command Window and run to visualize the graphical results*.
G. In the figures that are opened, use the pointer to select points of interest.
H. On the Command Window, use *clear;clc;* commands to clean the workspace and Command Window.

* The graphical results are sequentially shown, as follows:

i. All the measured air pressure signals.
ii. The measured voltage values related to the battery level for each performed test.
iii. The measured air pressure signals in the battery voltage value range of 12.3 to 11.5 V.
iv. The results of *MATLAB boxplot(X)* functions. The selected signals, input as *MATLAB boxplot(X)* functions, were those signals measured in the of battery voltage value range of 12.3 to 11.5 V.
v. The calculated amplitudes of the Fourier transforms.

Table S2 lists the values assigned to all histological parameters in the experimental (X_{Ei}) and control wound (X_{Ci}) specimens. It is important to note that in the *abs*., *phl*., *bact*., *s. c. p*. and *seq*. parameters, a value of "0" indicated the best wound healing process. Conversely, in the *s. t. gr*., *gr. depth*, *reepith*., *w. c. t*., and *b. m. d*. parameters, a value of "3" indicated the best wound healing process. The *cav. diam*. and *seq. diam*. parameters were measured in millimetres.

A Wilcoxon signed-rank test (α = 0.05) was performed on the experimental and control specimens for each histological parameter analysed by the pathologist. If the *p*-value was less than 0.05, it was considered a significant difference. As it is clearly visible from the last row of **Table S2**, the *b. m. d*. parameter was the only one to show a statistically significant difference (p = 0.021).

Table S2. Values assigned to all histological parameters in the experimental (X_{Ei}) and control wound (X_{Ci}) specimens.

	abs.		*phl.*		*bact.*		*s. t. gr.*		*gr. depth*		*reepith.*		*cav. diam.*		*s. c. p.*		*seq.*		*seq. diam.*		*w. c. t.*		*b. m. d.*	
	X_{E1}	X_{C1}	X_{E2}	X_{C2}	X_{E3}	X_{C3}	X_{E4}	X_{C4}	X_{E5}	X_{C5}	X_{E6}	X_{C6}	X_{E7}	X_{C7}	X_{E8}	X_{C8}	X_{E9}	X_{C9}	X_{E10}	X_{C10}	X_{E11}	X_{C11}	X_{E12}	X_{C12}
1st	1	0	0	0	1	0	1	0	3	3	1	1	25	11	3	1	1	1	5	5	1	1	3	2
2nd	0	1	0	0	0	0	0	1	2	3	1	1	17	15	0	1	1	1	5	10	1	1	2	2
3rd	0	0	1	0	0	0	2	1	2	2	1	1	5	6	2	2	1	0	3	0	1	1	2	2
4th	3	3	0	0	3	3	0	0	3	1	0	0	16	15	3	3	1	1	8	2	0	0	3	0
5th	1	3	0	1	0	3	1	0	2	2	0	0	10	16	2	2	1	2	5	5	0	0	3	2
6th	2	3	0	2	3	3	1	3	3	3	1	0	12	14	1	3	2	3	11	7	1	0	2	2
7th	0	0	0	0	1	0	2	1	2	2	1	1	7	10	1	1	2	1	3	2	1	0	3	2
8th	0	3	0	3	0	3	3	1	1	3	1	1	0	0	0	3	3	2	6	6	1	1	3	1
9th	0	3	0	0	0	2	3	0	3	2	1	0	5	5	2	2	1	1	6	5	1	0	3	1
10th	2	3	0	2	0	2	2	1	3	3	1	0	10	17	0	3	1	3	10	10	0	0	3	1
p	**0.058**		**0.134**		**0.139**		**0.178**		**>0.999**		**0.149**		**0.528**		**0.221**		**0.824**		**0.293**		**0.149**		**0.021**	

Tables S3–S12 show the contingency tables used to process the values assigned to the analyzed histological parameters *abs*., *phl*., *bact*., *s. c. p*., *seq*., *s. t. gr*., *gr. depth*, *reepith*., *w. c. t*. and *b. m. d*.

Table S3. *Contingency table related to the **abscess** parameter.*

		Without pneumatic device				*Total*
		0	1	2	3	
With pneumatic device	0	2	1	0	2	5
	1	1	0	0	1	2
	2	0	0	0	2	2
	3	0	0	0	1	1
Total		3	1	0	6	10

Result →→→→

Favourable case	6	60%
No difference	3	30%
Unfavourable case	1	10%
Total	10	100%

Table S4. *Contingency table related to the **phlegmon** parameter.*

		Without pneumatic device				*Total*
		0	1	2	3	
With pneumatic device	0	5	1	2	1	9
	1	1	0	0	0	1
	2	0	0	0	0	0
	3	0	0	0	0	0
Total		6	1	2	1	10

Result →→→→

Favourable case	4	40%
No difference	5	50%
Unfavourable case	1	10%
Total	10	100%

Table S5. *Contingency table related to the **bacteria** parameter.*

		Without pneumatic device				*Total*
		0	1	2	3	
With pneumatic device	0	2	0	2	2	6
	1	2	0	0	0	2
	2	0	0	0	0	0
	3	0	0	0	2	2
Total		4	0	2	4	10

Result →→→→

Favourable case	4	40%
No difference	4	40%
Unfavourable case	2	20%
Total	10	100%

Table S6. *Contingency table related to the **sinus tract granulation** parameter.*

		Without pneumatic device				*Total*
		0	1	2	3	
With pneumatic device	0	1	1	0	0	2
	1	2	0	0	1	3
	2	0	3	0	0	3
	3	1	1	0	0	2
Total		4	5	0	1	10

Result →→→→

Favourable case	7	70%
No difference	1	10%
Unfavourable case	2	20%
Total	10	100%

Table S7. *Contingency table related to the **granulation depth** parameter.*

		Without pneumatic device				*Total*
		0	1	2	3	
With pneumatic device	0	0	0	0	0	0
	1	0	0	0	1	1
	2	0	0	3	1	4
	3	0	1	1	3	5
Total		0	1	4	5	10

Result →→→→

Favourable case	2	20%
No difference	6	60%
Unfavourable case	2	20%
Total	10	100%

Table S8. *Contingency table related to the **septal cell proliferation** parameter.*

		Without pneumatic device				*Total*
		0	1	2	3	
With pneumatic device	0	0	1	0	2	3
	1	0	1	0	1	2
	2	0	0	3	0	3
	3	0	1	0	1	2
Total		0	3	3	4	10

Result →→→→

Favorable case	4	40%
No difference	5	50%
Unfavorable case	1	10%
Total	10	100%

Table S9. *Contingency table related to the* ***sequestration*** *parameter.*

		Without pneumatic device				***Total***
		**0**	**1**	**2**	**3**	
With pneumatic device	**0**	0	0	0	0	**0**
	**1**	1	4	1	1	**7**
	**2**	0	1	0	1	**2**
	**3**	0	0	1	0	**1**
Total		**1**	**5**	**2**	**2**	**10**

Result
→→→→

Favourable case	3	30%
No difference	4	40%
Unfavourable case	3	30%
Total	**10**	**100%**

Table S10. *Contingency table related to the* ***basophilic muscle decomposition*** *parameter.*

		Without pneumatic device				***Total***
		**0**	**1**	**2**	**3**	
With pneumatic device	**0**	0	0	0	0	**0**
	**1**	0	0	0	0	**0**
	**2**	0	0	3	0	**3**
	**3**	1	3	3	0	**7**
Total		**1**	**3**	**6**	**0**	**10**

Result
→→→→

Favourable case	7	70%
No difference	**3**	**30%**
Unfavourable case	0	0%
Total	**10**	**100%**

Table S11. *Contingency table related to the* ***reepithelization*** *parameter.*

		Without pneumatic device		***Total***
		**0**	**1**	
With pneumatic device	**0**	2	0	**2**
	**1**	3	5	**8**
Total		**5**	**5**	**10**

Result
→→→→

Favorable case	3	30%
No difference	7	70%
Unfavorable case	0	0%
Total	**10**	**100%**

Table S12. *Contingency table related to the* ***wound closure tract*** *parameter.*

		Without pneumatic device		***Total***
		**0**	**1**	
With pneumatic device	**0**	3	0	**2**
	**1**	3	4	**8**
Total		**5**	**5**	**10**

Result
→→→→

Favourable case	3	30%
No difference	7	70%
Unfavourable case	0	0%
Total	**10**	**100%**

Table S13–S14 show the descriptive statistic performedon the *cav. diam*. and *seq. diam*. parameters. All values are given in millimetres. The *cav. diam*. indicates the size of the residual cavity inside the wound, and the *seq. diam*. indicates the presence of necrotic tissue. Therefore, "Δ" values are the experimental specimen values subtracted from the control specimen values, since a lower value on the experimental specimen after treatment was expected. The pathologist stated a visible improvement could be observed if the calculated difference was at least 5 mm.

Table S13. *Table showing the difference in the values for the* ***cav. diam.*** *parameter in the control and experimental specimens.*

	cav. diam.		
	X_{E7}	X_{C7}	$\Delta = X_{C7} - X_{E7}$
1st	25	11	-14
2nd	17	15	-2
3rd	5	6	+1
4th	16	15	-1
5th	10	16	+6
6th	12	14	+2
7th	7	10	+3
8th	0	0	0
9th	5	5	0
10th	10	17	+2

Result →→→→

Median	***IRQ***
**0.5**	**3**

Table S14. *Table showing the difference in the values of the* ***seq. diam.*** *parameter in the control and experimental specimens.*

	seq. diam.		
	X_{E10}	X_{C10}	$\Delta = X_{C10} - X_{E10}$
1st	5	5	0
2nd	5	10	+5
3rd	3	0	-3
4th	8	2	-6
5th	5	5	0
6th	11	7	-4
7th	3	2	-1
8th	6	6	0
9th	6	5	-1
10th	10	10	0

Result →→→→

Median	***IRQ***
**-0.5**	**3**

In the results of the *cav. diam*. and *seq. diam*. parameters, only 10% of cases show a measured difference greater than or equal to the threshold value stated by the physicians (i.e. 5 mm).

Table S15 lists the discrete values assigned to all histological parameters in the experimental (X_{Ei}) and control wound (X_{Ci}) specimens. A discrete value of "0" indicated the occurrence of the best wound healing process while a discrete value of "3" represented the worst wound healing process.

A Wilcoxon signed-rank test (α = 0.05) was performed on the experimental and control specimens in each test. If the *p*-value was less than 0.05, it was considered a significant difference. The last row of **Table S15** clearly show that the 5th (p = 0.031), 8th (p = 0.021) and 10th (p = 0.013) tests had a statistically significant difference.

Table S15. Discrete values assigned to the histological parameters in the experimental (X_{Ei}) and control wound (X_{Ci}) specimens.

	1st		*2nd*		*3rd*		*4th*		*5th*		*6th*		*7th*		*8th*		*9th*		*10th*	
	X_{E1}	X_{C1}	X_{E2}	X_{C2}	X_{E3}	X_{C3}	X_{E4}	X_{C4}	X_{E5}	X_{C5}	X_{E6}	X_{C6}	X_{E7}	X_{C7}	X_{E8}	X_{C8}	X_{E9}	X_{C9}	X_{E10}	X_{C10}
abs.	1	0	0	1	0	0	3	3	1	3	2	3	0	0	0	3	0	3	2	3
phl.	0	0	0	0	1	0	0	0	0	1	0	2	0	0	0	3	0	0	0	2
bact.	1	0	0	0	0	0	3	3	0	3	3	3	1	0	0	3	0	2	0	2
s.t.gr.	0	1	1	0	1	2	0	0	0	1	3	1	1	2	1	3	0	3	1	2
gr. depth	3	3	3	2	2	2	1	3	2	2	3	3	2	2	3	1	2	3	3	3
reepith.	1	1	1	1	1	1	0	0	0	0	0	1	1	1	1	1	0	1	0	1
s.c.p.	3	1	0	1	2	2	3	3	2	2	1	3	1	1	0	3	2	2	0	3
seq.	1	1	1	1	1	0	1	1	1	2	2	3	2	1	3	2	1	1	1	3
w. c. t.	1	1	1	1	1	1	0	0	0	0	0	1	0	1	1	1	0	1	0	0
b.m.d.	2	3	2	2	2	2	0	3	2	3	2	2	2	3	1	3	1	3	1	3
p	**0.572**		**> 0.999**		**0.773**		**0.371**		**0.031**		**0.193**		**0.766**		**0.054**		**0.021**		**0.013**	

REFERENCES

1. Branca, F.P. La fisiologia della pressione sanguigna in *Fondamenti di Ingegneria Clinica: Volume1*. 167–173, ISBN: 978-88-470-0098-8 (Springer Science & Business Media, 2000).
2. D'Amico, A. & Di Natale, C. A contribution on some basic definitions of sensors properties. *IEEE Sens. J.* **1**(3), 183–190, doi: 10.1109/jsen.2001.954831 (2001).
3. Franco, S. Thermal Noise in *Design with operational amplifiers and analog integrated circuits* (3rd Ed.) 322–323, ISBN: 0-07-232084-2 (McGraw-Hill series in electrical and computer engineering, 2002).
4. D'Amico, A., Di Natale, C. & Sarro, P. M. Ingredients for sensors science. *Sens. Actuators, B* **207**, 1060–1068, doi: 10.1016/j.snb.2014.07.065 (2015).
